# Smartphone and Mobile App Use Among Physicians in Clinical Practice: Scoping Review

**DOI:** 10.2196/44765

**Published:** 2023-03-31

**Authors:** Mauricette Lee, Abu Bakar Shakran Bin Mahmood, Eng Sing Lee, Helen Elizabeth Smith, Lorainne Tudor Car

**Affiliations:** 1 Lee Kong Chian School of Medicine Nanyang Technological University Singapore Singapore; 2 Family Medicine and Primary Care Lee Kong Chian School of Medicine Nanyang Technological University Singapore Singapore; 3 National Health Group Polyclinics Singapore Singapore; 4 Department of Primary Care and Public Health School of Public Health Imperial College London London United Kingdom

**Keywords:** evidence-based medicine, specialist, general practitioners, GP, primary care physicians, mobile apps, consultants, surgeons, pediatricians, clinical care, mobile phone

## Abstract

**Background:**

Health care professionals are increasingly using smartphones in clinical care. Smartphone use can affect patient quality of care and clinical outcomes.

**Objective:**

This scoping review aimed to describe how physicians use smartphones and mobile apps in clinical settings.

**Methods:**

We conducted a scoping review using the Joanna Briggs Institute methodology and reported the results according to PRISMA-ScR (Preferred Reporting Items for Systematic Reviews and Meta-Analyses extension for Scoping Reviews) guidelines. We used the following databases in our literature search: MEDLINE, Embase, Cochrane Library, Web of Science, Google Scholar, and gray literature for studies published since 2010. An additional search was also performed by scanning the reference lists of included studies. A narrative synthesis approach was used.

**Results:**

A total of 10 studies, published between 2016 and 2021, were included in this review. Of these studies, 8 used surveys and 2 used surveys with focus group study designs to explore smartphone use, its adoption, experience of using it, and views on the use of smartphones among physicians. There were studies with only general practitioners (n=3), studies with only specialists (n=3), and studies with both general practitioners and specialists (n=4). Physicians use smartphones and mobile apps for communication (n=9), clinical decision-making (n=7), drug compendium (n=7), medical education and training (n=7), maintaining health records (n=4), managing time (n=4), and monitoring patients (n=2) in clinical practice. The Medscape medical app was frequently used for information gathering. WhatsApp, a nonmedical app, was commonly used for physician-patient communication. The commonly reported barriers were lack of regulatory oversight, privacy concerns, and limited Wi-Fi or internet access. The commonly reported facilitator was convenience and having access to evidence-based medicine, clinical decision-making support, and a wide array of apps.

**Conclusions:**

Smartphones and mobile apps were used for communication, medical education and training, clinical decision-making, and drug compendia in most studies. Although the benefits of smartphones and mobile apps for physicians at work were promising, there were concerns about patient privacy and confidentiality. Legislation is urgently needed to protect the liability of health care professionals using smartphones.

## Introduction

### Background

The use of smartphones has become increasingly indispensable [1]. This technology has revolutionized how people live, learn, work, communicate, and entertain themselves [2]. The use of smartphones among health care professionals is also widespread and affects the clinical care they provide [3]. Studies in various settings reported that most health care professionals use smartphones daily in their practice [4-8].

Smartphones and mobile apps offer an important and diverse set of clinical tools for health care professionals. They enable direct communication with colleagues and patients, instant access to medical knowledge, education, remote patient management, research, and digital diagnostics, to name a few [2]. However, the widespread adoption and use of smartphones in medical practice can affect the quality of care [9,10]. There are concerns about the impact of smartphones on professionalism, patient safety, and data confidentiality as well as the trustworthiness of sources accessed via smartphones [6-8,11-14].

The use of smartphones may vary between different groups of health care professionals and in different settings. For instance, some studies report that medical journal mobile apps are more commonly used by physicians than by nurses [15,16]. In contrast, medical calculators or drug compendium apps are used by both physicians and nurses [7,16]. Many medical mobile apps targeting health care professionals are available, and their number is growing. Studies have reported that the daily use of medical apps ranges from 1 to 20 minutes among physicians [7]. Knowing what types of apps are commonly used by various health care professionals can help discern their needs, guide the future evaluation of the quality of such apps, and inform the development of new apps.

### Objectives

A growing number of studies are exploring the use of smartphones among health care professionals as well as their experiences and perceptions of the role of smartphones in clinical care [4,6,7,9-11,13-15,17-22]. However, to date, there are no existing scoping reviews, systematic reviews, and research syntheses available on this topic. Our objective was to collate and describe how smartphones and mobile apps were used by physicians, specifically, specialists and family physicians, within clinical settings. We presented the barriers, facilitators, and opinions of physicians regarding smartphones and mobile apps.

## Methods

### Overview

A scoping review was conducted using the Joanna Briggs Institute methodology and reported according to PRISMA-ScR (Preferred Reporting Items for Systematic Reviews and Meta-Analyses extension for Scoping Reviews) [23]. The scoping review methodology consisted of five key steps: (1) identifying the research question; (2) identifying relevant studies; (3) study selection; (4) charting the data; and (5) collating, summarizing, and reporting the results. The study protocol was registered in the Open Science Framework registries [24].

### Step 1: Identifying the Research Question

This review aimed to collate and describe studies focusing on the use of smartphones among physicians. The overarching question for this scoping review was as follows: “How do physicians use smartphones in clinical practice?” More specifically, the research questions for this scoping review are as follows:

- What are the smartphone apps and features physicians access and why?
- How do physicians use their smartphones as an information source?
- What are physicians’ opinions of the impact of smartphones on clinical care?

### Step 2: Identifying Relevant Studies

We developed the MEDLINE (Ovid) search strategy collaboratively and iteratively with support from an experienced medical librarian. The search strategy was guided by relevant articles identified from previous manual searches, based on our research questions, and eligibility criteria (Multimedia Appendix 1). The same strategy was adopted to search for applicable studies in Embase, Cochrane Library, and Web of Science. Similar to previous studies, we also searched the reference lists of the included studies and gray literature in the first 10 pages of the search results in Google Scholar using the search terms in our search strategy and titles of the included studies [25,26]. Only studies published in the English language were included. Results were imported to EndNote 20 (Clarivate Analytics) [27].

The included studies in this review had to meet the inclusion and exclusion criteria presented in Textbox 1. We included studies published between January 2010 and January 2022 to capture data that aligned with the proliferation of smartphone ownership [28]. This review aimed to understand practicing physicians’, defined as specialists, and family physicians’ use of smartphones in clinical settings. Owing to the differences in training needs between medical trainees and nontrainees, medical trainees were excluded [29].

**Inclusion and exclusion criteria.**

**Inclusion criteria**

- Studies focusing on the use of smartphones among physicians defined as specialists and family physicians
- Studies exploring the use of mobile apps and the use of social media if this is done explicitly using smartphones (only if the motivation for use was physician driven)
- Studies focusing on personal smartphones, organizationally provided smartphones, or both
- Studies focusing on a mix of physicians if more than 50% of the physicians were specialists and family physicians
- Survey, observational, mixed methods, or qualitative studies
- Published between January 2010 and January 2022
- Printed in the English language

**Exclusion criteria**

- Studies that focused on patients, medical students, medical trainees, medical residents, house officers, health intervention, or medical education
- If the smartphones were implemented for research purposes
- Studies that focused on infection control of personal smartphones
- Editorials, opinion pieces, conference posters, and abstracts

### Step 3: Study Selection

**Figure 1:**
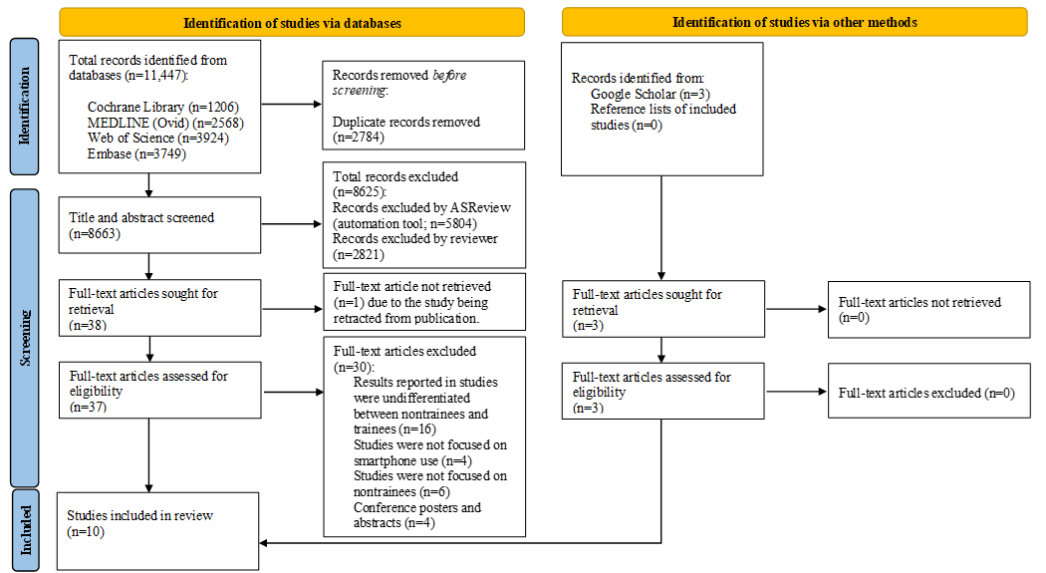
Flow diagram for the article selection process.

Studies were identified using the search criteria presented in Textbox 1. The search results from different electronic databases were combined in a single EndNote (Clarivate Analytics) library [27], and duplicate records were removed. The first reviewer (MLM) independently screened titles and abstracts on ASReview v1.0 (ASReview Lab) [30] to identify studies that potentially met the inclusion criteria. Only 33% of the titles and abstracts were screened. This was 1 rule that was predetermined and adhered to before screening commenced on ASReview [30]. This rule was set based on a study that found that 95% of eligible studies would be found after screening between 8% and 33% of studies on ASReview [31]. In parallel, a second reviewer (ABSBM) independently screened all titles and abstracts, including 33% of titles and abstracts that were screened by MLM on Covidence (Veritas Health Innovation) [32] to identify studies that potentially met the inclusion criteria. Disagreements on the included titles and abstracts were resolved through discussion between the first and second reviewers. Conflicts between the 2 reviewers were resolved through consensus, and when required, a third reviewer (Ahmad Ishqi Jabir) acted as an arbiter. In total, 2 reviewers (MLM and ABSBM) independently retrieved the full texts of the included titles and abstracts and read and assessed the studies against the eligibility criteria. Disagreements on the included full-text articles were resolved through discussion between the first and second reviewers. Conflicts between the 2 reviewers were resolved through consensus, and when required, a third reviewer (AIJ) acted as an arbiter. A total of 2 reviewers (MLM and ABSBM) independently extracted the data for each included study using a structured data extraction form. Figure 1 shows a flow diagram of the article selection process.

### Step 4: Charting the Data

After the screening process was completed, EndNote Library [27] was set up to share articles between the reviewers. A data charting form was created using Microsoft Excel and used to extract data from the included studies. The data extracted from each study included the name of the first authors, year of publication, title, aims of the study, study design, study location, study time frame, sample size, participant characteristics, type of smartphone used, and study findings (Multimedia Appendix 2 [5,22,33-40]). Adapted from previous studies by Lee and De Jong [41,42], the data extracted from the included studies were categorized into functions, benefits, and challenges of smartphones and mobile apps for health care professionals. We used coding frames from these studies, as they captured data that were aligned with the proliferation of smartphone ownership. The data charting form was piloted by 2 reviewers (MLM and ABSBM), with 4 studies either different in study designs or population specialties to ensure consistent, reliable, and efficient data extraction. Conflicts between the 2 reviewers were resolved through consensus, and when required, a third reviewer (AIJ) served as an arbiter.

### Step 5: Collating, Summarizing, and Reporting the Results

A comprehensive summary of the included studies, number of studies, study design, data collection methods, population types, and the aims of the study were presented. The use of smartphones was organized according to themes presented in a previous paper [41]. The framework we developed was aligned with all our research questions. The collated findings were ownership rates, type of mobile apps used, type of information sources used, type of websites accessed, use of smartphones for contact with colleagues and patients, and physicians’ experiences of using smartphones. Relationships between population characteristics and the use of smartphones (differences in the use of smartphones among physicians in primary and secondary care) were identified. A narrative synthesis of the findings without the use of a qualitative analysis program was presented.

## Results

### Search Findings

Database searches yielded 11,447 records, and another 3 records were retrieved from the gray literature source. After removing 2784 duplicates, 8663 titles and abstracts were screened. Title and abstract screening led to the exclusion of 8625 records, resulting in 40 full texts that needed to be assessed for eligibility. Of these, 30 articles were excluded, resulting in 10 studies for the review (Figure 1). We used a sensitive search strategy that aimed to retrieve all relevant research in this novel area and as such had a high number of citations initially. We then screened citations in parallel and independently to ensure the reliability of our screening.

### Study Characteristics

The 10 studies included in this review (Table 1 [5,22,33-40]) were conducted across 9 countries and published between 2016 and 2021. The study data collection time frames were reported in 4 studies [5,22,37,38], and data were collected between 2014 and 2019. Of the 10 included studies, 8 (80%) used surveys [5,22,33-38] and 2 (20%) used surveys with focus groups study designs [39,40]. A total of 3 studies recruited only general practitioners (GPs) [22,33,39], another 3 studies recruited only specialists [34,35,37], and 4 studies recruited both GPs and specialists [5,36,38,40]. The participants in the remaining studies were addressed as anesthetist consultants [34], pediatricians [35], specialists, or surgeons. Overall, 4 studies did not provide information on their clinical settings [5,33,35,36], 2 studies were conducted in hospitals [34,38], and the remaining studies were conducted in community health centers [22], large university surgical departments [37], rural practices [39], and health institutions [40]. The characteristics and details of the included studies on the use of smartphones and mobile apps by physicians are presented in Tables 1 and 2 (Multimedia Appendix 2). One study reported that physicians possessed more than 6 different work-related mobile apps on their smartphones [34]. Another study reported that most GPs had 1 to 3 medical apps, with very few owning more than 4 [22]. Only 1 study found that young GPs (aged <35 years) were more likely to own smartphones [22]. Another study found that younger physicians (aged ≤44 years) were less likely to allow their patients to communicate with them via the internet or phone, and they used medical apps more often [5]. One study reported that 10% (5/50) of physicians used organizationally provided smartphones, whereas the rest used personal smartphones for clinical use [38]. The results are presented in Tables 1 and 2 and are consistent with the PRISMA-ScR guidelines [43].

**Table 1:** Summary table of included studies (N=10).

Features			Study, n (%)
**Country**			
	Ireland	2 (20)	
	Austria	1 (10)	
	Australia	1 (10)	
	Canada	1 (10)	
	China	1 (10)	
	Cyprus	1 (10)	
	Ecuador	1 (10)	
	Sudan	1 (10)	
	Turkey	1 (10)	
**Year of publication**			
	2021	1 (10)	
	2020	2 (20)	
	2019	1 (10)	
	2018	2 (20)	
	2017	2 (20)	
	2016	2 (20)	
**Population type**			
	Only GPs^a^	3 (30)	
	Only specialists	3 (30)	
	GPs and specialists	4 (40)	
**Age group (years)**			
	25-35	1 (10)	
	36-45	4 (40)	
	46-66	3 (30)	
	>66	0 (0)	
	Not applicable^b^	2 (20)	
**Sex^c^**			
	Mostly male	5 (50)	
	Mostly female	3 (30)	
	Intersex	0 (0)	
	Not applicable	2 (20)	
**Type of smartphone used^c^**			
	Mostly Android	1 (10)	
	Mostly iPhone	3 (30)	
	Others	0 (0)	
	Not applicable	6 (60)	
**Most commonly reported frequency of smartphone use**			
	Daily	2 (20)	
	Weekly	1 (10)	
	Monthly	0 (0)	
	Sometimes	1 (10)	
	Rarely	0 (0)	
	Never	1 (10)	
	Not reported	5 (50)	
**Most commonly reported purpose of smartphone use**			
	Communication	9 (90)	
	Clinical decision-making	7 (70)	
	Medical education and training	7 (70)	
	Reference tools	7 (70)	
	Health record maintenance	4 (40)	
	Time management	4 (40)	
	Patient monitoring	2 (20)	

^a^GP: general practitioner.

^b^This information was not reported in this study.

^c^The number of studies was calculated based on the majority reported under sex and type of smartphone used. For instance, if a study reported more males than females being recruited, we counted it as “Mostly males.” Similarly, there are a number of studies on the type of smartphones used.

**Table 2:** Smartphones and mobile apps used by physicians.

Purpose of smartphone use		Examples of mobile apps and features used
Communication		WhatsApp [5,36,40], Google Hangout [40], Facebook [5,36], YouTube [36], SMS text messaging [36], email [22,36,37], voice calling [37], and instant messaging [33,37,39]
**Information seeking and management**		
	Clinical decision-making	Medscape [35,36,40], UpToDate [35,40], Nature [40], MedCalc [5,40], Das28 [40], Diagnostic assistance tools [38], Prognosis [40], Dxsarus [40], Laborwerte [5], Labormedizin Pocket [5]¸ and medical calculators [38]
	Health record maintenance	Enil [40], Meddata [40], E-nabiz [40], PACSapp [40], Acibadem [40], and coding and billing [42]
	Medical education and training	Twitter [34], Medscape [35,36,40], OrthoApp [40], Vcell [40], UpToDate [35,40], PubMed [36], and Nature [40]
	Reference tools	UpToDate [35], Medscape [35,36], Cepilaç [40], Diagnosia [5], Embryotox [5], Antibiotika (Thalhammer) [5], Arzneimittel Pocket [5], Eponyms [40], and PubMed [36]
**Clinical care**		
	Patient monitoring	Apple Health [37], Instant health rate [37], and Fitwell [37]
	Time management	Google Calendar [40]

### Communication

Of the 10 included studies, 7 (70%) reported the use of smartphones and mobile apps for communication [5,22,33,36,37,39,40]. One study reported that participants allowed their patients to contact them by phone and via web-based communication tools [5]. However, the study did not report on the smartphone features or apps used. Another study reported that smartphones allowed faster access to information, especially for communication among peers [40]. More than half of the physicians preferred using smartphones and mobile apps over other alternatives for communication with other physicians [36]. Of the 10 studies, 3 (30%) did not explicitly report how smartphones and mobile apps were used by physicians for communication [34,35,38]. One study reported using smartphones to contact other specialists for referrals or advice [34], another study reported using smartphones for communication [38], and the last study did not report on communication at all [35].

### Information Seeking and Management

Of the 10 included studies, 7 (70%) reported the use of mobile apps for medical education and training [5,34-38,40]. Of the 7 studies, 3 did not provide examples of mobile apps used by GPs and specialists in medical education and training [5,37,38]. Of the 3 studies, 1 reported that most physicians perceived that they were provided with reliable clinical content and continuing medical education when using mobile apps for medical education and training [5]. Another study reported that most surgeons felt that texting improved the educational experience of their trainees [37]. Of the 10 studies, 7 (70%) reported the use of mobile apps for clinical decision-making [5,22,34-36,38,40]. Of the 7 studies, 1 reported that anesthetists used mobile apps for clinical algorithms, clinical planning, and assessment [34]. Another study reported that mobile apps for disease diagnosis were used by GPs [22], but the identity of the mobile apps used was not provided in those 2 studies.

Frequent use of drug compendium apps was reported in 7 studies [5,22,34-36,38,40]. A total of 4 studies found that the most frequent type of medical apps used by physicians were drug compendium apps [5,22,34,40]. The other most common types of reference tools were the literature search portals [5,22,35,36,38] and medical literature [5,22,34-36]. These were followed by medical journals [34-36], medical news [5,35,36], and medical textbooks [5,40]. Of the 7 studies, 1 reported that anesthetists used mobile apps for drug compendium, prescription, and dosing, and academic journals [34]. However, no examples of the mobile apps used were provided in this study. Another study reported that the Diagnosia and Embryotox medical apps were most often used [5]. Physicians were less familiar with medical apps such as Antibiotika (Thalhammer) from Germany, MedCalc (United States), Laborwerte (Germany), Arzneimittel Pocket (Germany), and Labormedizin Pocket (Germany) [5].

Of the 10 included studies, 4 (40%) reported the use of mobile apps for health record maintenance to access local appointment systems developed by their local health ministry [5,34,38,40]. Of the 4 studies, 1 reported the types of smartphone apps used by anesthetists, including apps for billing and accessing patient results and those that allowed access to hospital electronic medical records [34]. Another study reported that Enlil (Turkey), a national hospital management information system, was the most used app [40]. It was followed by other medical health recording systems from Turkey, such as Meddata, E-nabiz, PACSapp, and Acibadem [40].

### Clinical Care

The use of mobile apps for time management was reported in 40% (4/10) of the included studies [22,34,35,40]. Only 1 study reported Google Calendar as the most used app among GPs, followed by the default mobile calendar and appointment app [40]. The other 3 studies did not provide examples of mobile apps used for time management [22,34,35].

Only 20% (2/10) of studies reported the use of mobile apps for patient monitoring [38,40]. According to Sezgin et al [40], patient-monitoring apps were the least prevalent among all mobile apps. Examples of the types of patient-monitoring smartphone apps provided in that study were pedometers, calorie trackers, and heart rate and information tracker tools (cardiograph) [40]. The study also reported that patients had been using the tracking apps and sharing them with their physicians [40]. Teferi et al [38] reported using an app to document the procedures for patient monitoring.

### Smartphone Features Used and Web Access by Physicians

Of the 10 studies, only 1 (10%) reported the use of built-in features in the smartphone, such as torchlight, stopwatch, and camera [34]. The same study also reported the use of a smartphone to distract pediatric patients [34]. However, the study did not report on how the smartphone features were used by physicians.

A total of 2 studies reported that physicians used nonmedical apps more frequently than medical apps [22,42]. These nonmedical apps were used by physicians for calendar [22] and web access [22,42]. Overall, 4 studies reported on the use of smartphones for web access [22,35,38,40]. However, 3 of the 4 studies neither reported the reasons for physicians to access the internet nor stated the websites that were accessed [22,38,40]. Web browsers and search engines were also used for medical information searches, sometimes outperforming medical apps [40].

### Barriers to the Use of Smartphones and Mobile Apps by Physicians

#### Overview

In addition to depicting the use of smartphones and mobile apps by physicians, many studies have described the factors that prevented physicians from using smartphones. This review found a wide variety of barriers to the use of smartphones and mobile apps by physicians, including the infringement of patient privacy and confidentiality, lack of regulatory oversight, negative impact on physician-patient and collegial relationships, quality concerns, limited Wi-Fi or internet access, lack of workplace integration, and lack of smartphone savviness.

#### Infringement of Patient Privacy and Confidentiality

The potential confidentiality breach of patient privacy was the most common barrier to the use of smartphones and mobile apps by physicians [33,36,37,39,40]. Only one study reported on privacy concerns, which included the fear of sending the message to the wrong person or number and the uncertainty around the receipt of the messages [33]. The lack of security and control over the apps’ content were also perceived to be risks related to the infringement of patient privacy and confidentiality when using smartphones and mobile apps for communication at work [40]. However, the study did not report the names of these apps.

#### Lack of Regulatory Oversight

The included studies also addressed barriers to the regulation of smartphones and mobile apps used by physicians [33,37,39,40]. One study reported that only 27% of GPs have a written text policy for texting patients [33]. GPs who used texts always documented patient consent, and when texting medically sensitive information, they always obtained specific consent [33,39]. In the hospital setting, some physicians were unaware that they had any organizational policy on the use of smartphones [34] and sharing patient information via text messages [37]. Most surgeons in 1 study agreed that texting patient-related information should be regulated by a hospital policy (74%) or legislation (57%) [37].

#### Negative Impact on Physician-Patient and Collegial Relationships

Several studies in this review referenced barriers related to professional relationships. Hofer and Haluza [5] reported that employees were not allowed to use their smartphones at work, as it was found to be disruptive to the relationship with patients during consultation. It was also reported that, among consultant anesthetists with more than 3 years of experience as a consultant, up to 27% agreed that their smartphone was a distraction from their work [34]. However, none of those with less than 3 years in post believed that their smartphones were a distraction from their work [34]. In addition to distraction, GPs found that text messaging increased patient anxiety [39]. Some physicians also reported feeling uncomfortable using smartphones in front of patients [40].

#### Quality Concerns

Another reported barrier was concern about the quality of the information provided by medical apps [5,40]. For example, physicians expected professional organizations to inform evidence-based medical apps, including assessing the quality of medical information in the medical apps recommended by the organization [5].

#### Limited Wi-Fi or Internet Access

In total, 2 studies reported limited internet access as a barrier [5,36]. Physicians mentioned that they would use many more apps if smartphone reception was better in the hospital [5]. Hence, they suggested that the availability of an offline version of an app is important [5].

#### Lack of Workplace Integration

Sezgin et al [40] also raised the issue of the lack of extensive use of smartphones and mobile apps in the hospital system. This has been demonstrated by the lack of interoperability between the use of smartphones and other hospital devices [40].

#### Lack of Smartphone Savviness

Only one study reported the lack of advanced skills as a barrier to the use of smartphones and mobile apps [40]. For example, some physicians indicated that they were not aware and unsure of the appropriate apps that could be used to help them with their daily clinical tasks. Their lack of knowledge on smartphone use prevented them from using it in clinical settings.

### Facilitators for the Use of Smartphones and Mobile Apps by Physicians

#### Overview

Numerous studies have reported on the facilitators for the use of smartphones and mobile apps by physicians. Facilitators included convenience and access to evidence-based medicine and clinical decision-making support. One study reported that smartphones and mobile apps were useful for conducting research. However, the authors did not elaborate further [36]. The user-friendliness of medical apps was perceived to facilitate the ease of use of mobile apps [5].

#### Convenience

Physicians used smartphones and mobile apps primarily for convenience [5,33,34,36,37,39,40]. For example, flexible communication channels [5,33,34,36,37,40] as well as a selection of powerful apps to accomplish a variety of tasks at work were readily available [5,36]. Portability [36,41], rapid access to information [37], and multimedia resources [5,36,41] were also examples of convenience.

#### Access to Evidence-Based Medicine and Clinical Decision-making Support

Access to various evidence-based and clinical decision-making support mobile apps was highlighted as a facilitator in this review [5,34,40]. The evidence-based medical mobile apps included apps for medical education and training and reference tools, as listed in Table 2.

## Discussion

### Summary of Key Findings

According to the studies included in this review, physicians primarily use smartphones and mobile apps for communication, medical education and training, clinical decision-making, and accessing the drug compendium. Medscape was frequently mentioned as a medical app used for information gathering. WhatsApp has been widely reported as a nonmedical app used for physician-physician and physician-patient communication. The most common barriers reported in the included studies were the risk of infringing on patient privacy and confidentiality, lack of regulatory oversight, limited Wi-Fi or internet access, the lack of extensive use of mobile apps in the hospital system, and the lack of smartphone savviness. The most common facilitators reported in the included studies were the availability of having flexible communication methods, easy access to evidence-based medicine, clinical decision-making support, availability of mobile app choices to accomplish many different purposes at work, and portability.

We found that physicians are more likely to use smartphones for work-related purposes because of the increasing availability of mobile apps. Prior studies showed that only 13% of physicians used their smartphones to watch web-based videos weekly for professional purposes, and continuing medical education activities were the most frequently viewed content [44]. However, this review found that studies frequently reported the daily and weekly use of smartphones and mobile apps by physicians [5,22,40]. In addition, smartphones and mobile apps were widely used not only for medical education and training but also for communication, clinical decision-making, and reference tools. We found mixed views regarding the use of smartphones for work-related purposes as a distraction for physicians [5,34]. For instance, physicians believe that using a smartphone during a consultation could negatively affect the patient-physician relationship [5]. This finding is consistent with a recent systematic review on the effect of web-based information-seeking behavior on the physician-patient relationship [45]. Another study found a correlation between the number of years of experience as a specialist and whether smartphones were perceived as a distraction [34]. Younger physicians tended to use smartphones more and were more likely to accept them in the workplace [34]. This was also found to be consistent with a recent systematic review of distraction with smartphones during nursing care [46]. Future research should conduct a review on the distraction of smartphones from physicians in the clinical setting and perhaps derive a precise estimate of the effect that smartphone distraction has on clinical care outcomes.

Our review identified some challenges to the use of smartphones and mobile apps by physicians. First, we found that physicians were unaware of their hospital’s policy [41] on the use of smartphones at work. Only a minority of GPs had written a text policy for texting patients [32]. As a result, while our review found that most GPs who used text messaging always documented patient consent when texting medically sensitive information [33], there remains the potential for a breach in confidentiality. Although previous studies have suggested that the use of strong authentication mechanisms helps to mitigate the risks of a breach [47,48], we found evidence that not all physicians had their smartphones encrypted or password protected, and others were unsure whether their smartphones were encrypted [37].

Physicians are increasingly using instant messaging tools, such as WhatsApp, Facebook, and Google Hangout, for physician-physician and physician-patient communications. However, using social media at work may result in the mingling of personal and hospital data. Most social media tools mentioned in our review are not Health Insurance Portability and Accountability Act compliant, which aims to protect patient privacy and ensure the integrity of sensitive medical information [49]. Despite the absence of Health Insurance Portability and Accountability Act–specific regulations for smartphones and apps, some organizations have developed recommendations and guidelines for mobile security measures [49-53]. A previous study [47] suggested that educating health care professionals about the available hospital policy on the use of smartphones at work could be useful in implementing the policy. However, our review revealed a lack of direction for ideal smartphone use at work. Consequently, it might be helpful to have a policy or legislation that provides comprehensive guidance on authentication, access control, chain of responsibility, data ownership, allowed devices, acceptable use, training, and noncompliance with the use of smartphones and mobile apps [47,54]. Compliance with the legislation of smartphone use at work should be considered in the future during the appraisal process of health care professionals.

This review found that physicians use evidence-based medical apps because they provide instant access to evidence. One example of such an app is the evidence-based point-of-care information summaries [5,34,40]. Point-of-care information summaries are defined as medical compendia specifically designed to deliver predigested, rapidly accessible, comprehensive, periodically updated, and evidence-based information (and possibly guidance) to clinicians [55]. Our review found that Medscape and UpToDate were the most commonly reported evidence-based point-of-care information summaries apps. However, health care organizations lack information on the use of evidence-based medical apps [5]. They were unaware of the reliability of evidence-based information provided by medical apps [5]. To ensure quality and safety, the use of medical apps must undergo rigorous evaluation, validation, and development of best practice standards [40]. Therefore, as a means of mitigating the use of non–evidence-based information in clinical practice, future research should assess the quality of evidence within medical apps to support health care professionals to be more confident when using such apps for practice. In addition, the findings from such research may inform policy on the audit and regulation of medical apps.

There were some limitations when conducting this scoping review. As 8 (80%) of the 10 studies used quantitative methods such as surveys to gather data, deep descriptions and examples to provide an in-depth understanding of smartphones and mobile app use were limited [5,22,33-38]. In addition, only English-language studies were included in this narrative synthesis. Although our classification of data was determined through detailed analysis, team discussions, and consensus, there may be themes that we have overlooked. However, as our comprehensive analysis was based on a commonly used framework on smartphone use by health care professionals, missing out on themes may have been minimized [41].

### Implication of the Findings

Physicians use smartphones and mobile apps for communication, clinical decision-making, drug compendium, medical education and training, maintaining health records, managing time, and monitoring patients in clinical practice. However, we found several gaps related to the use of smartphones and mobile apps by physicians at work. These gaps are the lack of regulatory oversight either at a hospital or at a government level, that is, the need to address concerns about the risks of infringement of patient privacy and confidentiality when using smartphones and mobile apps for communication of patient information. There is a need to identify medical apps that provide reliable clinical information and nonmedical apps that can be used for communication by physicians at work. We also found that the use of smartphones differs in different subgroups, such as participants of different ages, sexes, and work experience. Therefore, there may be possible implications on the association of the characteristics of participants with the use of smartphones and mobile apps. Future studies should explore the associations between smartphone use with clinical practice. Future research should also provide more information about smartphone use in clinical practice, including whether smartphones were used for work-related or personal purposes, how smartphone features and apps were used, and how health care professionals communicate using smartphones.

### Conclusions

Our review found literature reporting on the use of smartphones and mobile apps for communication, medical education and training, clinical decision-making, and drug compendia. Challenges related to the use of smartphones and mobile apps include the lack of patient privacy and confidentiality and regulatory oversight. The benefits of smartphones and mobile apps for physicians at work include the availability of having flexible communication methods and mobile app choices to accomplish many different purposes at work, easy access to evidence-based medicine and clinical decision-making support, and portability. Physicians commonly use Medscape and WhatsApp mobile apps. Future research should address patient privacy issues, as well as legislation related to smartphone and mobile apps in clinical practice.

## Appendix

Multimedia Appendix 1 Search strategy.

Multimedia Appendix 2 Characteristics of the included studies on the use of smartphones and mobile apps for physicians.

## Abbreviations

GP: general practitioner
PRISMA-ScR: Preferred Reporting Items for Systematic Reviews and Meta-Analyses extension for Scoping Reviews
