# Supplementary material for: Smartphone and Mobile App Use Among Physicians in Clinical Practice: Scoping Review
Source: JMIR Mhealth Uhealth. 2023 Mar 31;11:e44765. doi: 10.2196/44765 (PMC10131676; doi:10.2196/44765)
Supplement: Multimedia Appendix 1 [file mhealth_v11i1e44765_app1.docx]

Multimedia Appendix 1: Search strategy
