# Supplementary material for: Smartphone and Mobile App Use Among Physicians in Clinical Practice: Scoping Review
Source: JMIR Mhealth Uhealth. 2023 Mar 31;11:e44765. doi: 10.2196/44765 (PMC10131676; doi:10.2196/44765)
Supplement: Multimedia Appendix 2 [file mhealth_v11i1e44765_app2.docx]

Multimedia Appendix 2: Characteristics of the included studies on the use of smartphones and mobile apps for physicians

| Authors (year), study design, study location/country | Mean age (%) | Gender | Target population | Type of smartphones used | Study aims | Frequency of smartphones and mobile apps use | Purpose of smartphones and mobile apps use |
| --- | --- | --- | --- | --- | --- | --- | --- |
| Hofer and Haluza (2019) [5], survey, Austria. | 25–35 (0%)  36–45 (100%)  46-66 (0%)  >66 (0%) | Female - 55%  Male - 45% | 151 General Practitioners (GPs) and specialist physicians | NA | - To obtain a comprehensive picture of the readiness to use medical apps from the perspective of Austrian practitioners. | Daily (39%)  Never use or may not use daily (61%) | - Communication - Medical education and training - Patient monitoring - Health record maintenance - Clinical decision-making - Reference tools |
| Liu et al. (2016) [22], survey, six community health centres from the six main districts of Hangzhou city, Eastern China. | 25–35 (0%)  36–45 (100%)  46-66 (0%)  >66 (0%) | Female - 54%  Male - 46% | 125 GPs | Android (48%)  iPhone (49%)  Other mobile phones (3%) | - To identify the extent to which GPs own mobile phones and which types. - To evaluate how often they use apps to acquire medical information and support clinical decisions. - To investigate the types and frequency of apps used. | Daily (36%)  Monthly (0%)  Never used (8%)  Rarely (24%)  Weekly (32%) | - Communication - Clinical-decision making - Reference tools |
| Quinlan et al. (2018) [33], survey, members of the Irish College of GPs. | 25-45 (30%)  46-66+ (70%) | Female - 52%  Male - 48% | 536 GPs | NA | - To address knowledge deficit and ascertain the extent, benefits, risks, barriers and clinical role of text messaging in Irish general practice. | NA | - Communication |
| Perkins et al. (2020) [34], survey, a large tertiary private metropolitan hospital in Melbourne, Australia. | 25–35 (0%)  36–45 (48%)  46-66 (50%)  >66 (2%) | Female - 29%  Male - 71% | 113 Consultant anaesthetists | Android (9%)  iPhone (91%) | - To evaluate and characterise the use of smartphones in clinical situations by Australian anaesthetists. - To explore their perception of whether their smartphone was a facilitator of, or barrier to, their clinical practice. | About half the time (13%)  Always (27%)  Most of the time (24%)  Sometimes (36%) | - Communication - Information management - Medical education and training - Time management - Patient monitoring - Health record maintenance - Clinical decision-making - Reference tools |
| Hadjipanayis et al. (2016) [35], survey, members of the National Paediatric Association Registry of Cyprus. | 25–35 (0%)  36–45 (0%)  46-66 (100%)  >66 (0%) | Female - 47%  Male - 53% | 75 Paediatricians | Android (49%)  iPhone (47%)  Other mobile phones (4%) | - To present the current prevalence of smartphone ownership among Cypriot paediatricians and the patterns of their use in everyday clinical practice. | Browsing internet:   - 60 mins/day (33%) - > 30 mins/day (39%) - Between 31-60 mins/day (27%) | - Information management - Medical education and training - Time management - Clinical decision-making - Reference tools |
| Cherrez-Ojeda et al. (2020) [36], survey, cross-sectional, Ecuador | 25–35 (0%)  36–45 (100%)  46–66 (0%)  >66 (0%) | Female - 46%  Male - 53%  Undisclosed – 1% | 640 GPs and specialists | NA | - To conduct an objective assessment of the frequency of use, perceptions, and barriers of Information and Communication Technologies among physicians in mainstream medical practice. | NA | - Communication - Medical education and training - Clinical decision-making - Reference tools |
| Firdouse et al. (2018) [37], survey, a large university surgical department in Toronto, Canada. | 25–35 (0%)  36–45 (53%)  46–66 (47%)  >66 (0%) | Female - 36%  Male - 61%  Undisclosed – 3% | 62 Academic surgeons | Android (5%)  iPhone (75%)  Other mobile phones (20%) | - To understand texting practices among staff surgeons. | NA | - Communication - Medical education and training - Time management |
| Teferi et al. (2021) [38], survey cross-sectional, in 5 referral hospitals (Gondar University, Felege Hiwot, Debre Markos, Dessie, and Debre Birhan referral hospitals) in Amhara region, Ethiopia. | NA | NA | 290 GPs and specialists | NA | - To assess smartphone medical app use and associated factors among physicians working at referral hospitals of the Amhara region, Ethiopia. | NA | - Communication - Medical education and training - Patient monitoring - Health record maintenance - Clinical decision-making - Reference tools |
| Leahy et al. (2017) [39], survey, focus-group, urban and rural practices in the south-west of Ireland. | NA | NA | 389 GPs | NA | - To assess the extent, growth, perceived risks and benefits of text messaging by GPs to communicate with patients, and assess patients’ attitudes towards receiving text messages from their GP. | NA | - Communication |
| Sezgin et al. (2017) [40], survey, focus group, health institutions in Turkey. | 25–35 (53%)  36–45 (36%)  46–66 (11%)  >66 (0%) | Female- 44%  Male - 56% | 147 GPs and specialists | NA | - To understand physicians’ awareness of mobile health apps and their intentions to use these apps in medical practice. | Daily (17%)  Never used (1%)  Monthly (28%)  Rarely (0%)  Weekly (54%) | - Communication - Information management - Medical education and training - Time management - Patient monitoring - Health record maintenance - Clinical decision-making - Reference tools |
| NA: not applicable; this information was not reported in the study. | | | | | | | |
